# Predicting gene regulatory links from single-cell RNA-seq data using graph neural networks

**DOI:** 10.1093/bib/bbad414

**Published:** 2023-11-20

**Authors:** Guo Mao, Zhengbin Pang, Ke Zuo, Qinglin Wang, Xiangdong Pei, Xinhai Chen, Jie Liu

**Affiliations:** Science and Technology on Parallel and Distributed Processing Laboratory, National University of Defense Technology, deya, 410073 Changsha, China; Science and Technology on Parallel and Distributed Processing Laboratory, National University of Defense Technology, deya, 410073 Changsha, China; Science and Technology on Parallel and Distributed Processing Laboratory, National University of Defense Technology, deya, 410073 Changsha, China; Science and Technology on Parallel and Distributed Processing Laboratory, National University of Defense Technology, deya, 410073 Changsha, China; Science and Technology on Parallel and Distributed Processing Laboratory, National University of Defense Technology, deya, 410073 Changsha, China; Science and Technology on Parallel and Distributed Processing Laboratory, National University of Defense Technology, deya, 410073 Changsha, China; Science and Technology on Parallel and Distributed Processing Laboratory, National University of Defense Technology, deya, 410073 Changsha, China; Laboratory of Software Engineering for Complex System, National University of Defense Technology, deya, 410073 Changsha, China

**Keywords:** graph neural network, link prediction, graph convolutional network, gene regulatory networks (GRNs)

## Abstract

Single-cell RNA-sequencing (scRNA-seq) has emerged as a powerful technique for studying gene expression patterns at the single-cell level. Inferring gene regulatory networks (GRNs) from scRNA-seq data provides insight into cellular phenotypes from the genomic level. However, the high sparsity, noise and dropout events inherent in scRNA-seq data present challenges for GRN inference. In recent years, the dramatic increase in data on experimentally validated transcription factors binding to DNA has made it possible to infer GRNs by supervised methods. In this study, we address the problem of GRN inference by framing it as a graph link prediction task. In this paper, we propose a novel framework called GNNLink, which leverages known GRNs to deduce the potential regulatory interdependencies between genes. First, we preprocess the raw scRNA-seq data. Then, we introduce a graph convolutional network-based interaction graph encoder to effectively refine gene features by capturing interdependencies between nodes in the network. Finally, the inference of GRN is obtained by performing matrix completion operation on node features. The features obtained from model training can be applied to downstream tasks such as measuring similarity and inferring causality between gene pairs. To evaluate the performance of GNNLink, we compare it with six existing GRN reconstruction methods using seven scRNA-seq datasets. These datasets encompass diverse ground truth networks, including functional interaction networks, Loss of Function/Gain of Function data, non-specific ChIP-seq data and cell-type-specific ChIP-seq data. Our experimental results demonstrate that GNNLink achieves comparable or superior performance across these datasets, showcasing its robustness and accuracy. Furthermore, we observe consistent performance across datasets of varying scales. For reproducibility, we provide the data and source code of GNNLink on our GitHub repository: https://github.com/sdesignates/GNNLink.

## INTRODUCTION

A gene regulatory network (GRN) is a graphical representation of the regulatory interdependencies between regulatory factors and target genes, where the target genes play a role in controlling the transcriptional state of a cell [1]. The analysis of biomedical networks, such as GRNs, holds significant potential in advancing our understanding of complex human diseases, aiding in their prevention, diagnosis and treatment, as well as facilitating the identification of potential drug targets for therapeutic development [2, 3].

The rapid advancement of Single-cell RNA-sequencing (scRNA-seq) technology has generated an exponential growth of single-cell gene expression data [4]. Consequently, there is an urgent need to develop computational approaches that can efficiently extract and integrate essential information from these large datasets to uncover potential gene interdependencies. scRNA-seq data captures the dynamics of gene expression levels across individual cells during cellular differentiation, providing insights into cellular heterogeneity. However, the presence of noise and sparsity resulting from dropout events presents significant challenges in inferring GRNs from scRNA-seq data in computational biology [5–7]. In recent years, researchers have proposed computational methods to address the limitations associated with single-cell expression data and reveal the underlying regulatory interdependencies behind complex cellular phenotypes from a genomic perspective [8, 9]. These methods can be broadly classified into three main approaches: information theory-based methods, machine learning-based methods and deep learning-based methods.

Information theory-based methods, also known as relevance methods, have gained widespread popularity in the field [10–12]. The approach of this class assumes that genes within the same group tend to display similar expression patterns during physiological processes. The basic way is by calculating the correlation between genes, where higher correlation values indicate a higher likelihood of interaction. The advantages of these methods is their relatively low computational complexity and minimal sample size requirements, which allows the construction of large networks from small amounts of data. However, the limitation of this approach is that the correlations are bidirectional and therefore the inferred gene network is undirected [12, 13]. This limitation implies that information regarding causality and regulatory dependencies between genes may not be accurately captured. In practical terms, it means that while these methods can identify associations between genes based on co-expression patterns, they may not distinguish between upstream and downstream regulatory interdependencies.

For example, Shachaf *et al*. [14] developed an algorithm that leverages k-nearest neighbor (kNN) mutual information (MI) estimation to enhance the accuracy of estimating MI for bivariate and trivariate Gaussian distributions. This approach effectively reduces errors in estimating MI and has demonstrated significant improvements in reconstructing GRNs. Specht *et al*. [15] developed the LEAP method by calculating Pearson correlations on fixed size time windows with different lags, and the method finally takes the maximum Pearson correlation for all lagged values. Aibar *et al*. [16] propose SCRIBE, an information-theoretic method, to construct GRNs based on the mutual information between the past state of a regulator and the current state of a target gene. The method leverages computational techniques to measure the strength of the regulatory interdependencies and employs the contextual relevance likelihood algorithm [17] to eliminate edges corresponding to indirect effects.

The machine learning-based approach focuses on fitting gene expression data using machine-learning computational methods and data structures [10]. The most representative are regression methods [18], which are highly interpretable and can identify the regulation direction, so that the inferred regulation network is a directed graph. However, there are huge data sample requirements for this approach, and the machine learning models need samples to be trained. Therefore for small samples of data, the construction of GRN is not effective [9, 12]. These limitations can restrict the applicability and accuracy of GRN inference when dealing with insufficient data.

For instance, Huynh-Thu *et al*. [19] introduced GENIE3, a Random Forest (RF)-based approach that achieved first place in the DREAM5 In Silico Web Challenge in 2010. GENIE3 decomposes the prediction of intergenic regulatory networks into multiple regression problems, where each regression problem aims to predict the expression pattern of a target gene based on the expression patterns of other genes. Li *et al*. [20] introduced a framework called LogBTF, which binaryizes single-cell gene expression data into Boolean values (0 or 1). They then constructed a Boolean threshold network model by combining Boolean threshold functions with logistic regression coefficients to infer GRNs. Woodhouse *et al*. [21] proposed SCNS, a boolean logic rule-based method that utilizes single-cell gene expression data collected over a period of time. SCNS calculates boolean logic rules to capture the progression and transformation of cell states from the initial to the later stages. Gao *et al*. [22] proposed a ridge regression approach SINCERITIES to construct GRNs. SINCERITIES utilizes changes in the expression of transcription factors (TFs) in one time window to predict how the expression distribution of target genes will change in the subsequent time window. These machine learning methods treat the problem of reconstruction networks (GRNs) as a regression problem and demonstrate the effectiveness and versatility of utilizing machine learning algorithms to extract meaningful patterns from gene expression data.

In recent years, the field of computational biology has witnessed the emergence of several deep learning frameworks, inspired by the remarkable success of deep learning in computer vision [23–25]. Deep learning-based methods aim to process raw biological data and transform it into a format that can be effectively interpreted by specific deep learning models. The disadvantage of these types of deep learning is that it causes losing data during the data transformation process, which may affect the quality and applicability of the inferred network [26, 27].

For example, Yuan *et al*. [27] develops a convolutional neural network (CNNC) method. This method encodes gene expression data into a feature matrix, and the feature matrix is then classified by a convolutional neural network (CNN). However, the CNNC method converts each gene pair into a normalized empirical probability function matrix containing a large number of genes is a tedious task. And insufficient data transformation will not be able to effectively training the CNN model. Kishan *et al*. [28] propose a gene network embedding (GNE) method, which uses network topology and gene expression profile data to predict gene interdependencies using a multilayer perceptron (MLP). However, GNE encounters difficulties in fully understanding the gene topology due to limited information on the intricate gene dependencies. Zhao *et al*. [29] developed DGRNs, a hybrid deep learning framework that combines recurrent neural networks (RNN) and CNN to reconstruct GRNs from single-cell gene expression data. The disadvantages of this method are the high complexity and the need for temporal information in the gene expression data. Chen *et al*. [6] propose GENELink, which transforms the regulatory problem into a linkage prediction problem. The method uses incomplete a priori networks and gene expression data to predict potential regulatory interdependencies using graph attention networks (GAT). However, an obvious drawback of the model is the lack of negative sample data and the high computational complexity.

**Figure 1:**
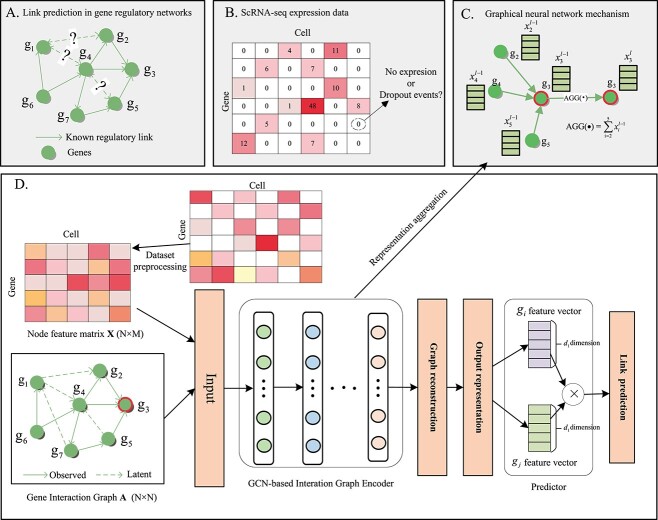
Overview of GNNLink framework. (**A**) We consider the inference of GRNs by supervised methods as a linkage prediction problem, where the objective is to identify potential edges based on existing ones. (**B**) ScRNA-seq expression data imputation. (**C**) The demonstration of learning node feature, where AGG($\cdot $) denotes an aggregation operation that accumulates the features of other nodes connected to a specific node, such as node 3. D. The general structure of the GNNLink model. It involves three main steps. Firstly, the raw data are preprocessed to prepare it for further analysis. Secondly, the node features are learned, capturing important features and information regarding the genes. Finally, the interaction graph is reconstructed for link prediction, with the regulatory interdependencies between genes $i$ and $j$ represented by the dot product between them.

To address the above problem, we propose a novel method called GNNLink, which focuses on inferring GRNs through link prediction. Our approach formulates the task as predicting potential links in the network, given a set of genes and some observed interdependencies (as depicted in Figure 1D). Our model comprises several key components. Firstly, we utilize biological data from databases to construct initial GRNs. Subsequently, we preprocess the single-cell gene expression data to extract gene features. Secondly, we employ a graph convolutional network (GCN)-based interaction graph encoder that effectively captures dependencies among genes, extracts informative gene features and facilitates their transfer to downstream tasks. Finally, we will predict the gene-to-gene regulatory scores based on the learned gene features. For the gene regulatory linkage prediction task, we perform a large number of experiments. The results demonstrate the effectiveness of the gene features learned by our GNNLink model, which leads to improved performance and reduced training time. In general, our main contributions are summarized as follows:

- GNNLink formulates the problem of inferring GRNs as a link prediction task. This approach aims to predict potential interdependencies between genes in the network, leveraging both known and inferred interdependencies.
- GNNLink introduces a GCN-based interaction graph encoder. This component efficiently captures and maintains the dependencies between genes in the network. It extracts valuable gene features from both the network structure and gene expression data, facilitating various downstream tasks, including similarity measurement and causal inference between gene pairs.
- Experimental results demonstrate that GNNLink effectively trains gene features, leading to improved model performance. The model not only enhances prediction accuracy but also reduces training time, making it a valuable tool for gene regulatory linkage prediction tasks.

## MATERIALS AND METHODS

### Datasets

The data used in this paper are scRNA-seq datasets of seven cell types provided by BEELINE [9], which specifically include human embryonic stem cells (hESC [27]), human mature hepatocytes (hHEP [30] ), mouse dendritic cells (mDC [31]), mouse embryonic stem cells (mESC [32]), mouse hematopoietic stem cells of the erythroid lineage (mHSC-E), mouse hematopoietic stem cells with a granulocyte-monocyte lineage (mHSC-GM) and mouse hematopoietic stem cells with a lymphoid-like lineage (mHSC-L). The scRNA-seq datasets can be downloaded from Gene Expression Omnibus using the following accession numbers: GSE81252 (hHEP), GSE75748 (hESC), GSE98664 (mESC), GSE48968 (mDC) and GSE81682 (mHSC [33]). We also used cell type-specific ChIP-seq [34], non-cell type-specific transcriptional regulatory network ChIP-seq [35] and functional interdependencies from the STRING database [36] as ground truth networks. For the mESC dataset, a Loss/Gain of Function (LOF/GOF) ground truth network is also available [37]. The specific statistics are shown in Table 1.

**Table 1:** The statistics of single-cell transcriptomic datasets and four ground-truth networks with TFs and 500 (1000) most-varying genes

Cell types	Cells	STRING			Non-specific ChIP-seq		
		TFs	Genes	Density	TFs	Genes	Density
hESC	759	343(351)	511(695)	0.024(0.021)	283(292)	753(1138)	0.016(0.014)
hHEP	426	409(414)	646(874)	0.028(0.024)	322(332)	825(1217)	0.015(0.013)
mDC	384	264(273)	479(664)	0.038(0.032)	250(254)	634(969)	0.019(0.016)
mESC	422	495(499)	638(785)	0.024(0.021)	516(522)	890(1214)	0.015(0.013)
mHSC-E	1072	156(161)	291(413)	0.029(0.027)	144(147)	442(674)	0.022(0.020)
mHSC-GM	890	92(100)	201(344)	0.040(0.037)	82(88)	297(526)	0.030(0.029)
mHSC-L	848	39(40)	70(81)	0.048(0.045)	35(37)	164(192)	0.048(0.043)
Cell types	Cells	Cell-type-specific ChIP-seq			Loss Of Function (LOF)/Gain Of Function (GOF)		
		TFs	Genes	Density	TFs	Genes	Density
hESC	759	34(34)	815(1260)	0.164(0.165)	-	-	-
hHEP	426	30(31)	874(1331)	0.379(0.377)	-	-	-
mDC	384	20(21)	443(684)	0.085(0.082)	-	-	-
mESC	422	88(89)	977(1385)	0.345(0.347)	34 (34)	774 (1098)	0.158(0.154)
mHSC-E	1072	29(33)	691(1177)	0.578(0.566)	-	-	-
mHSC-GM	890	22(23)	618(1089)	0.543(0.565)	-	-	-
mHSC-L	848	16(16)	525(640)	0.525(0.507)	-	-	-

### Method overview

Link prediction leverages existing network information and properties of nodes to infer the presence of new or hidden links in the network by drawing on the already existing network structure. In the field of link prediction, graph neural networks (GNN) have gained considerable attention in recent years [38–40]. Notably, Ye *et al*. [41] proposed a method called gene graph convolutional neural network (GCNG) that leverages GNN for learning node features by aggregating features from neighboring nodes. GCNG infers gene interdependencies related to cell-cell communication, using spatial single-cell expression data.

### The GNNLink framework

As shown in Figure 1, GNNLink is a supervised deep learning framework, which uses scRNA-seq gene expression data and generic GRN as input. In a GRN, the edges symbolize the regulatory interdependencies between TFs and target genes. These dependencies are directed, originating from the TFs and pointing towards the target genes. They represent various types of regulatory dependencies, including both direct and indirect regulations, as well as activations or inhibitions [9]. These dependencies are typically derived from extensive cell sequencing data such as ChIP-seq data, as well as literature sources [35]. Our work focuses on training gene features that can leverage gene features information and gene interaction networks to predict downstream tasks of genes. The training model framework proposed in Figure 1(D). It consists of three main parts: (i) raw data preprocessing, (ii) interaction graph encoder based on GCN and (3) GRN reconstruction.

### Preprocessing of raw data

To preprocess the raw scRNA-seq data, we adopted an established method [9] due to the vastness and redundancy of the data. Our preprocessing steps aimed to filter out low-expressed genes and focus on genes with significant variability in expression. Initially, we removed genes expressed in less than 10% of cells, eliminating low-expressed genes from further analysis. Next, we calculated the variance and $P$-value for each gene’s expression vector. Specifically, we focused on genes with $P$-values below 0.01 after applying Bonferroni correction [9]. The gene expression levels are normalized by logarithmic transformation. Upon applying the aforementioned operations to the single-cell expression data, we derived a feature matrix $X \in{\Re ^{N \times M}}$, where $N$ represents the number of genes and $M$ represents the number of cells. To assess the efficacy of our computational approach across networks of varying sizes, we employed a ranking strategy based on gene variance, following the methodology proposed by Pratapa *et al*. [9]. We select the highly variable TF and the top 500 and 1000 genes with the greatest variability in expression, which will generate 14 different datasets.

### GCN-based interaction graph encoder

To derive the initial features of the genes, we apply preprocessing operations to the raw single-cell expression data. Subsequently, we utilize GCN-based interaction graph encoders to learn gene features by leveraging the underlying structure of the gene interaction graph.

Let us denote the prior network as $G = \{V,\xi \}$, where $V \in{\Re ^{N}} $ is the set of $N$ nodes and $\xi $ is the set of edges. In the context of the graph encoder, the primary objective is to learn the features of each node, denoted as $v_{i}$, in $G$ by iteratively aggregating the features of its neighboring nodes. Formally, the $l$th layer of the GCN-based graph encoder can be described as follows: 


(1) \begin{eqnarray*} h_{i}^{l} = AGGREGATE(\{ h_{j}^{l - 1}:{v_{j}} \in{\mathrm{\mathcal{N}}_{i}}\} ) \end{eqnarray*}

In the context of the graph encoder, the features of node $v_{j}$ at the ($l$-1)th layer are denoted as $h_{j}^{l - 1}$. The first-hop neighbors of node $v_{i}$ in the network, including node $v_{i}$ itself, are represented as $\mathcal{N}_{i}$. To update the feature of node $v_{i}$ at the $l$th layer, an aggregator function, denoted as AGGREGATE(.), plays a crucial role in the graph encoder by incorporating the features of neighboring nodes. It is a versatile component that can be defined using various graph neural architectures, including the popular GCN.

GCNs have have emerged as a powerful tool for analyzing graph-structured data, demonstrating remarkable effectiveness across various research domains. In light of this, we leverage GCNs in our study to acquire gene features through the integration of first-order neighbor feature data, characterizing this procedure as a GCN layer. To begin, we assume that each node in the network is self-connected. This assumption allows us to define a normalized adjacency matrix, denoted as $\widetilde A$. The matrix $\widetilde A$ is computed as $\widetilde A = {{\text{D}}^{ - \frac{1}{2}}}A{D^{\frac{1}{2}}}$, where $A$ represents the adjacency matrix of the graph. The element $A_{ij}$ in $A$ indicates the presence of an edge between nodes $v_{i}$ and $v_{j}$. The diagonal matrix $D$ is constructed such that its diagonal elements ${D_{ii}}$ are equal to the sum of the corresponding row in $A$. In this study, we utilize GCN as an aggregation function to update node features. Given the node feature matrix $H^{(l-1)}$ at layer ($l - 1$), we compute the feature matrix $H^{(l)}$ at layer $l$ of the GCN using the following formula: 


(2) \begin{eqnarray*} H^{(l)} = ReLU(\widetilde A{H^{(l - 1)}}{W^{(l - 1)}} + {b^{(l - 1)}}) \end{eqnarray*}

where $ReLU$ is the activation function and $H^{l-1}$ denotes the model’s output at layer $l-1$. It’s important to note that $H^{(0)}$ corresponds to the input feature matrix $X$. The trainable weight matrix and bias vector are denoted as $W^{(l-1)}$ and $b^{(l-1)}$, respectively. After the $L$th layer of GCN, the output of the last layer is adopted as the final gene features denoted as $H$, which is a matrix of size $N \times d_{1}$. Here, $N$ represents the number of genes, and $d_{1}$ represents the dimension of the gene features.

### Link prediction

Upon obtaining the gene feature matrix, it becomes valuable for predicting potential gene regulatory dependencies. Straightforwardly, our GNNLink model conducts dot product operations on the feature vectors between genes to generate comprehensive gene regulatory dependencies, as described below: 


(3) \begin{eqnarray*} R = ReLU(H{H^{T}}) \end{eqnarray*}

where $ReLU$ is the activation function.The score matrix $R$ represents the reconstructed GRNs, where each element of the matrix corresponds to the regulatory action score of a gene pair.

### Model optimization

Building upon the aforementioned methodology for obtaining the score matrix $R$ of the reconstructed GRN using the input interaction network $G$ and its adjacency matrix $A$, we derive the loss function as follows: 


(4) \begin{eqnarray*} \ell = \sum\limits_{(i,j) \in{\Omega^ +} \cup{\Omega^ -}} {\Phi (R(i,j),A(i,j)) + \lambda \left\| \Theta \right\|}_{\text{F}}^{2} \end{eqnarray*}

where $\Omega ^ +$, $\Omega ^ - $ denote the positive and negative sample sets used for model training, respectively. The parameter matrix of the GNNLink model is represented as $\Theta $, and a weighting factor $\lambda $ is used to control the impact of the $\Theta $ parameter. To optimize the model, we employ the mean square error (MSE) loss, denoted as $\Phi (.)$, which quantifies the difference between the predicted gene regulation scores and the actual dependencies in the datasets. We utilize the Adam optimizer [42] to iteratively update the model parameters until convergence, enabling us to obtain the gene regulation score matrix $R$. the magnitude of the $R_{ij}$ score between the $i$th and $j$th genes indicates the strength of the regulatory dependencies between them. A higher score implies a stronger regulatory connection. Note that when the gene features is trained, the parameters of the encoder are also updated simultaneously. In this work, we incorporate a negative sampling strategy to train our model effectively. This strategy involves sampling negative examples that do not exhibit regulatory dependencies, which helps the model learn to distinguish between positive and negative instances and improves its overall performance.

## RESULTS

### Experimental setting

We record directed regulatory dependencies between gene pairs in a benchmark network called positive samples and label them as 1. Conversely, gene pairs not present in the benchmark network are called negative samples and labeled as 0. According to the network density in Table 1, the number of negative samples significantly exceeds that of positive samples. We randomly select 3/5 of the known gene pairs as the positive training set. The remaining 1/5 and 1/5 are allocated as the positive test set and positive validation set, respectively. Considering the high dimensionality of the negative set, in practice, we use hard negative samples during training to enhance the learning process of node features, as suggested by Zhu *et al*. [43]. The concept of hard negativity is incorporated into the training dataset by pairing each positive gene pair ($g_{1}$,$g_{2}$) with a negative gene pair ($g_{1}$,$g_{3}$), where both pairs share the same gene $g_{1}$. The selection of hard negative samples utilizes a uniform random negative sampling strategy from the remaining negative samples. We ensure the proportion of positive and negative samples in each dataset aligns with the network density. Our model is trained on the training set, while the hyperparameters are iteratively optimized using the validation set. This iterative optimization process enables us to fine-tune the model’s performance and improve its ability to capture the underlying regulatory dependencies within the gene network.

Due to the imbalance of positive and negative samples, we use two well-known performance evaluation metrics, namely the area under the receiver operating characteristic curve (AUROC) and the area under the precision-recall curve (AUPRC). For the training of the GNNLink model, we set the training period to 200 iterations and utilize a learning rate of 0.005. The GNN architecture consists of two layers, with hidden layer sizes set to 256 and 128, respectively. Although these parameter values are determined empirically, we perform a parametric analysis on the number of layers ($L$) and weight factor ($K$) in the GCN-based graphical encoder in subsequent sections. The results provided in this subsection are based entirely on the predictions of the test set. To ensure a fair and unbiased evaluation, we maintain consistency by utilizing identical training and validation sets for all supervised methods. This approach allows us to compare the performance of each model on the same test set, eliminating potential variations introduced by different data splits. For further information regarding the specific parameter settings of the GNNLink model, please refer to Supplementary Notes 1.2.

### Baseline methods

To assess the effectiveness of our model in predicting GRNs, we compare our model against six state-of-the-art baseline methods commonly used for inferring GRNs, as follows:

- GENELink [6] proposes a graph attention network approach to infer potential GRNs.
- GNE [28] proposes an MLP approach to encode both gene expression profiles and network topology for the prediction of gene dependencies.
- CNNC [27] proposes to infer GRNs using deep convolutional neural networks.
- DeepDRIM [44] is a supervised deep neural network that utilizes images to represent the expression distribution of joint gene pairs. These images serve as the input to the neural network, which performs binary classification tasks using both target TF-gene pair images and images of potential neighbors.
- GRN-transformer [45] is a weakly supervised learning method that utilizes axial transformers to infer cell type-specific GRNs from scRNA-seq data and generic GRNs.
- GENIE3 [19] is a random forest-based machine learning method that constructs GRNs based on regression weight coefficients. the method won first place in the DREAM5 In Silico Network Challenge held in 2010.

We have selected the above models based on their alignment with the research problem, their status as state-of-the-art baseline methods, the diversity of their approaches, and the availability of reproducible code. For the sake of consistency and comparability, we utilize the default parameters as provided in the original implementations of all baseline methods. Detailed information regarding the execution settings and running specifics of each baseline method can be found in Supplementary Notes 1.1.

### Parameter analysis

**Figure 2:**
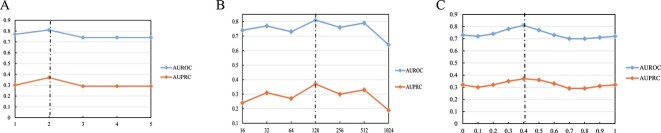
Parameter sensitivity analysis. Parameter sensitivity analysis for GNNLink model in terms of (**A**) number of layers of encoder $L$, (**B**) dimension of representation $d_{1}$ and (**C**) weight factor $\lambda $. The average performance measured by AUROC and AUPRC among seven datasets. Abbreviations: AUROC, area under the receiver operating characteristic curve; AUPRC, area under the precision-recall curve.

The performance of our model is influenced by several crucial parameters, including the dimensionality of the gene feature (denoted as $d_{1}$) at the final layer, the number of GCN layers ($L$) and the weight factor ($\lambda $) in the GCN-based interaction graph encoder. To assess the model’s effectiveness in predicting GRNs, we conduct an evaluation on seven benchmark datasets consisting of cell-type-specific networks. We perform fine-tuning by varying the parameter values and thoroughly analyze their impact on GRN prediction. The average performance across the seven datasets is depicted in Figure 2, while Supplementary Figures S1 and S3 provide additional insights.

The number of layers ($L$) plays a crucial role in determining the set of neighboring features in the GCN-based interaction graph encoder. To evaluate the effect of this parameter, we systematically vary $L$ from 1 to 5, with a step size of 1. Analysis of Figure 2(A) and Supplementary Figure S1 reveals that setting $L$ to 2 yields the highest average AUROC and average AUPRC values. Interestingly, we observe diminishing returns when using more layers, potentially due to overfitting and smoothing issues. Next, we investigate the influence of the representation dimension ($d_{1}$) in our model. We explore $d_{1}$ values from a range of 16, 32, 64, 128, 256, 512, 1024. The results depicted in Figure 2(B) and Supplementary Figure S1 indicate that extreme values of $d_{1}$, whether too large or too small, are not optimal for model performance. Notably, the best average AUROC and AUPRC scores are achieved when $d_{1}$ is set to 128, suggesting an optimal balance between representation capacity and complexity. Finally, the weighting factor $\lambda $ controls the contribution of known GRNs. We explore values of $\lambda $ from 0 to 1 with a step size of 0.1. $\lambda = 0$ means that only single-cell gene expression data are available as input data for the model. $\lambda = 1$ means that only a priori gene regulatory dependencies are used in the model. Figure 2(C), Supplementary Figure S1 and Supplementary Figure S3 illustrate that GNNLink exhibits little sensitivity to the choice of $\lambda $. Consequently, we set $\lambda $ to 0.4 in our experiments.

### Performance on benchmark datasets

To assess the effectiveness of the GNNLink method, we use the dataset described in Table 1 and evaluate its performance using AUROC and AUPRC. We compare the GNNLink method with six benchmark algorithms, including GENELink [6], GNE [28], CNNC [27], DeepDRIM [44], GRN-Transformer [45] and GENIE3 [19]. These algorithms have demonstrated state-of-the-art performance on benchmark datasets, as confirmed by the evaluation of the GENELink method.

**Figure 3:**
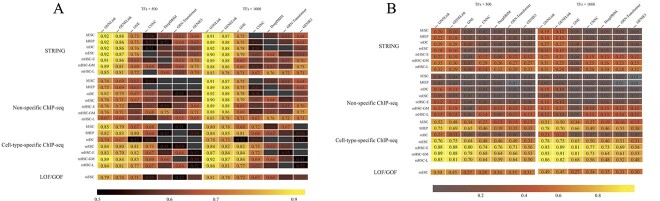
Summary of the GRN prediction performance in the AUROC metric (**A**) and the AUPRC metric (**B**). Our evaluation is conducted on seven single-cell RNA sequencing (scRNA-seq) datasets, each comprising four ground-truth networks. The scRNA-seq datasets consist of significantly varying transcription factors (TFs) and the 500 (left) or 1000 (right) most-varying genes. (A) The AUROC values in the heatmap represents the average performance across 50 independent calculations for each dataset. The black squares indicate instances where the performance is poorer than random predictors, as denoted by an AUROC value below 0.5. (B) The AUPRC values in the heatmap also are averaged over 50 calculations for each dataset.

The results are shown in Figure 3. Overall, the GNNLink method demonstrates superior performance compared to all other baseline methods when applied to the scRNA-seq dataset. Specifically, the AUROC values obtained by GNNLink are approximately 9.2% and 18.3% higher on average than the second-best methods, namely GENELink and GNE, respectively. Considering the AUPRC metric displayed in Figure 3B, GNNLink achieves the best prediction performance on 93.18% (41 out of 44) of the benchmark datasets. And it improves at least 4% compared to the second best method (GENELink) on about 40.9% (18 out of 44) of the benchmark dataset. In addition, GNNLink outperforms GRN-Transformer, CNNC and DeepDRIM by a wide margin in most benchmark tests. In all benchmark tests, GNNLink improves 3.59, 13.97 and 17.59% in the average AUPRC metric over GENELink, GNN and GENIE3, respectively.

To assess the performance of the GNNLink model under different imbalanced ratios, we conducted experiments on the mESC dataset using the LOF/GOF network, which has a positive-to-negative ratio of 0.187. Specifically, we chose the positive-to-negative ratios of 1:5, 1:6 and 1:7 in the vicinity of 0.187, and the experimental results are shown in Supplementary Fig. S2. All seven methods demonstrated a positive correlation between the positive-to-negative link ratio and model performance. Notably, when the ratio was 1:5, GNNLink achieved the highest performance with an AUROC of 0.83 and an AUPR of 0.52. However, when the ratio was 1:7, all seven methods showed a significant drop in performance, with some methods failing to learn a suitable model.

### Robustness of GNNLink

Our model relies on a GCN-based interaction graph encoder to effectively captures the expression feature patterns of genes. We further explore the impact of training data size on the performance of our supervised model, i.e. whether features can be extracted efficiently. We conduct experiments on seven distinct datasets containing cell- type-specific ground truth sources of GRNs. To compare the robustness of the different methods, we randomly subsample 10, 20, 30, 40 and 50% of the supervised training data.

**Figure 4:**
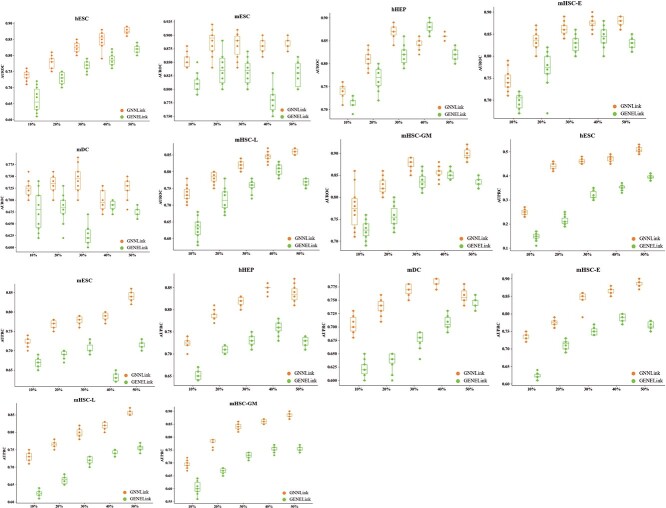
The average performance measured by AUROC and AUPRC for different numbers of training samples. Performance of GENELink with a wide range of training set sizes from 10 to 50% on seven benchmark datasets with cell-type-specific networks. Each method is run 10 times on each dataset. Abbreviations: AUROC, area under the receiver operating characteristic curve; AUPRC, area under the precision-recall curve.

Figure 4 illustrates the performance of the GENELink method across a range of training dataset sizes, from 10 to 50%, in cell-type-specific GRNs. As anticipated, the performance of all methods in making predictions typically shows enhancement with an increase in the amount of supervised training data. Notably, both the GNNLink and GENELink methods reach a steady state in performance when 40% of the training data is used. Compared to the GENELink method, GNNLink also achieves performance improvements (on average) of 4.93 (AUROC) and 9.94% (AUPRC). These improvements highlight the effectiveness of GNNLink in leveraging even small training datasets to achieve decent outcomes in GRN inference.

To investigate the impact of changing the ratio of training, validation and test sets on the performance and robustness of the GNNLink model, we conducted experiments on the hHEP dataset with the cell-type-specific ChIP-seq network. The dataset was divided into 10 parts and evaluated the model’s performance using training, validation and test set ratios of 8:1:1 and 4:3:3, as presented in Supplementary Table S2. Our results showed that the 4:3:3 ratio did not provide sufficient training data, resulting in poor performance for all models. However, the 8:1:1 and 3:1:1 ratios had minimal impact on model performance and were not influenced by the proportion of positive samples. Consequently, we adopted the traditional machine learning split ratio of 3:1:1, as it did not affect the model’s robustness and performance.

To further investigate the stability of the GNNLink model against data perturbations, we added Gaussian noise with different standard deviations ($\sigma $ = 0.01, 0.02, 0.05) to the single-cell gene expression data in the hESC, hHEP, mDC and mESC datasets, which contained cell-type-specific GRNs. These noisy datasets were then used for training, validation and testing. We compared the performance of the GNNLink model with six other models under varying noise ratios, and the results are shown in Supplementary Table S1. We observed that all models showed a declining trend in performance after noise was added. These experiments indicate that hyperparameter optimization is more important for the model itself than for the dataset, and that the GNNLink model exhibits good stability and robustness, effectively avoiding the issue of overfitting.

### Impacts of single-cell data imputation

Single-cell gene expression data are shown in Figure 1(B), where zero elements may arise due to missed detection of gene expression, resulting in artificially assigned zero expressions for numerous genes. To address this issue, several efficient computational methods have been developed in recent years to estimate the missing zero entries in scRNA-seq data. Here, we use DeepImpute [46] to impute zero entries in the raw scRNA-seq dataset. Next, we evaluate the impact of imputation using AUROC and AUPRC on seven raw data sets with cell type-specific networks. Because of the random characteristics of deep learning algorithms, such as initializing random weights, it can give different results by training the same network with the same data.

**Figure 5:**
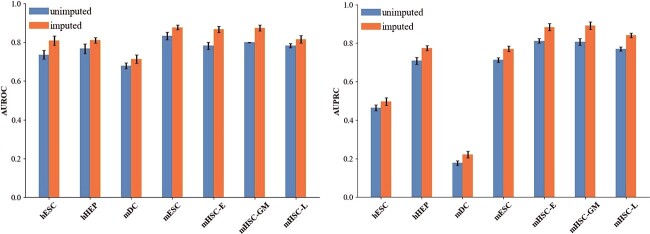
The performance of GNNLink on imputed and unimputed benchmark datasets with cell-type-specific networks. Average GRN inference performance of seven scRNA-seq datasets, i.e. AUROC (left panel) and AUPRC (right panel). We use DeepImpute [46] to impute the zero-entries in the raw seven benchmark datasets.We report the mean $\pm $ variance over 10 repeats. Abbreviations: AUROC, area under the receiver operating characteristic curve; AUPRC, area under the precision-recall curve.

The results of the GNNLink method run 10 times on each dataset are shown in Figure 5. Compared to the raw data, the GNNLink method shows the best prediction performance using the DeepImpute [46] method to impute the zero elements of the raw single-cell data. Overall, the GNNLink performance using the DeepImpute method for scRNA-seq data improved by an average of 5.55 and 6.12% in in the evaluation metrics of AUROC (Figure 5, left) and AUPRC (Figure 5, right), respectively. These results provide experimental evidence that the presence of zero values in scRNA-seq data significantly affects the prediction accuracy of the GNNLink method. By leveraging existing imputation methods to address sparsity and technical noise in scRNA-seq data, such as imputing missing values in scRNA-seq expression data, the accuracy of inferring GRNs can be improved.

### Computational complexity

We investigate the running time of each method on seven scRNA-seq datasets. Table 2 shows the average running time of each method on different sizes of seven single-cell datasets with cell type-specific networks. Our method has the shortest run time on the TFs+500 genes and TFs+1000 genes scRNA-seq datasets (please refer to Table 1). GENIE3 has the second best runtime on the two different scaled datasets owing to the multi-threaded parallel computation used by GENIE3 to improve efficiency. It is worth mentioning that when considering the computational complexity of these inference algorithms, our method proves to be more efficient and stable in inferring GRNs from datasets of various sizes, including both small and large datasets. Moreover, our algorithm exhibits a relatively shorter and more efficient running time.

**Table 2:** The average running time of each method.

Average running time	GNNLink	GENELink [6]	GNE [28]	CNNC [27]	DeepDRIM [44]	GRN-Transformer [45]	GENIE3 [19]
TFs+500 genes	4.4 s	4 min 45 s	6 min 12 s	1 h 10 min	3 h	5 min 34 s	1 min 4 s
TFs+1000 genes	8.59 s	15 min 06 s	34 min 08 s	18 h 41 min	20 h 7 min	27 min 41 s	8 min 12 s

## DISCUSSION AND CONCLUSION

ScRNA-seq technologies provide a valuable opportunity to delve into regulatory mechanisms at the single-cell level. While various analytical tools have emerged for scRNA-seq data analysis, their primary focus remains on addressing cellular heterogeneity. However, GRNs hold a central position in comprehending cellular regulatory mechanisms, making the precise inference of GRNs from scRNA-seq data imperative. ScRNA-seq, despite its power, encounters challenges like dropout events leading to zero-inflated data. Although existing imputation methods prove useful, they may inadvertently introduce bias and unpredictable noise. Additionally, the integration of TF-DNA binding data, such as ChIP-seq, can furnish valuable prior regulatory information. Nonetheless, this data resource remains accessible for only a fraction of TFs, and TF-DNA binding motifs may be incomplete. Therefore, there is a pressing need to develop a robust tool for predicting regulatory links by leveraging incomplete observed TF-gene dependencies together with single-cell gene expression data. By doing so, we can advance our understanding of cellular regulatory mechanisms and overcome the limitations posed by dropout events and the availability of TF-DNA binding data.

In this work, we present a novel supervised training framework called GNNLink, which utilizes GNNs to predict potential dependencies from scRNA-seq data and gene network topology. First, we perform preprocessing operations on the raw datasets. Meanwhile, we utilize the a priori GRN as the initial gene interaction graph. Second, we design a GCN-based interaction graph encoder. This encoder takes the interaction graph and initial gene expression levels as inputs, allowing us to aggregate the features of nodes (genes) and their neighboring nodes in the graph. Finally, the gene feature is used for dot product operations on the reconstructed GRN task. Through comprehensive analysis of scRNA-seq datasets from seven different cell types and four real networks, we demonstrate the superior performance of our GNNLink model. It significantly outperforms state-of-the-art models in terms of accuracy and efficiency.

Although our model has achieved good performance, there are still some limitations that need to be addressed. Specifically, the node features employed for reconstructing GRNs may not be optimal, particularly considering the sparsity of known ground truth networks. Additionally, our use of a uniform distribution strategy in negative sampling, although not critically impacting model training, has inherent limitations. GNNLink assumes equal importance among all negative samples, disregarding potentially influential rare and significant negative samples.

To overcome these limitations, we will explore two key avenues in future research. First, integrating self-supervised learning techniques can enhance the learning of node (gene) features, and this can be further bolstered by incorporating additional domain knowledge, such as scATAC-seq and single-cell methylation data. Second, alternative negative sampling strategies should be investigated to better capture the intricacies of the data and improve the model’s ability to learn from negative samples. The current uniform sampling approach may result in a lack of diversity in negative samples, potentially introducing bias and diminishing the model’s overall effectiveness in capturing complex data dependencies. Acknowledging and addressing these limitations will advance the accuracy and applicability of the GNNLink model in GRN inference.

The GNNLink model can be effectively employed to construct GRNs in various types of data, including diverse cell RNA-seq datasets and plant cells, provided that the input data aligns with the model’s specific characteristics and requirements. This adaptability is attributed to the model’s training data and its ability to generalize well to novel datasets. Even in scenarios where obtaining a priori GRN information for non-specific cells is challenging, our GNNLink model remains valuable for GRN inference. In such cases, we can employ existing computational methods, such as information theory-based approaches [10–12], to estimate GRNs as prior information for single-cell data. Subsequently, the GNNLink model utilizes both single-cell data and the inferred prior GRN as input sources to predict potential gene regulatory dependencies. By leveraging these combined sources, our model provides valuable insights into gene regulatory dependencies, even in the absence of specific prior knowledge.

**Key Points**

- GNNLink is a novel supervised framework that utilizes graph neural networks (GNNs) to predict potential gene dependencies from scRNA-seq data and gene network topology.
- GNNLink incorporates a graph convolutional network (GCN)-based interaction graph encoder, which aggregates node (gene) features and captures dependencies among genes in the interaction graph.
- GNNLink outperforms state-of-the-art models in both accuracy and efficiency when evaluated on seven scRNA-seq datasets containing four ground truth networks.
- GNNLink exhibits excellent robustness and accuracy while maintaining a lower computational complexity, even when confronted with datasets of varying scales.

## Supplementary Material

oup-authoring-suppl_bbad414

## Data Availability

All the codes and datasets are available at https://github.com/sdesignates/GNNLink.

## Author Biographies

**Guo Mao** received her PhD degree from National University of Defense Technology. her current research interests are in bioinformatics, computational biology, and network analysis.

**Xinhai Chen** received his PhD degree from National University of Defense Technology. His current research interests are in intelligent computing, high performance computing, and deep learning.

**Xiangdong Pei** received his PhD degree from National University of Defense Technology. His primary research interests include accelerating artificial intelligence computations and industrial computing.

**Zhengbin Pang** is a professor in College of Computer, National University of Defense Technology, China. His research interests range across high-speed interconnect, heterogeneous computing, and high performance computer systems.

**Qinglin Wang** is an associate processor with National Key Laboratory of Parallel and Distributed Computing, National University of Defense Technology. His research interests include high-performance computing, VLSI signal processing, and machine learning.

**Ke Zuo** is a senior data scientist and professor who applies AI and machine learning to solve problems in healthcare, program optimization, and big data.

**Jie Liu** is a professor in Science andTechnology on Parallel and Distributed Processing Laboratory, National University of Defense Technology, China. His research interest includes parallel algorithm and high performance computing.
