# Supplementary material for: Predicting gene regulatory links from single-cell RNA-seq data using graph neural networks
Source: Brief Bioinform. 2023 Nov 20;24(6):bbad414. doi: 10.1093/bib/bbad414 (PMC10661972; doi:10.1093/bib/bbad414)
Supplement: oup-authoring-suppl_bbad414 [file oup-authoring-suppl_bbad414.pdf]

Predicting gene regulatory links from single-cell RNA-seq data using graph neural networks

October 25, 2023

1 Supplementary Notes

1.1 Baseline methods used in this study

In this study, we compare the performance of GNNLink with six different baseline methods, including GENELink[1], GNE[2], CNNC[3], DeepDRIM[4], GRN-Transformer[5], and GENIE3[6]. We obtained the code for CNNC[3] and DeepDRIM[4] from their respective github repositories(https://github.com/xiaoyeye/CNNC and https://github.com/jiaxchen2-c/DeepDRIM) and used the default hyperparameters provided by the original studies. However, we removed the scRNA-seq preprocessing step (log-transform) in the 'image feature generation' process, as all scRNA-seq data used in this study were preprocessed by the BEELINE benchmark[7] and BoolODE [7]. Considering previous studies that used cell-type-specific GRN as training labels, we further fine-tuned the training strategy to avoid overfitting. This led to improvements of 0.6% and 4.5% for CNNC and DeepDRIM on the BEELINE benchmark compared to the default training strategy.

For GENIE3[6], GENELink[1], GNE[2], and GRN-Transformer[5], we downloaded the code from their respective github repositories(https://github.com/Murali-group/Beeline, https://github.com/zpliulab/GENELink, https://github.com/kckishan/GNE, https://github.com/HantaoShu/GRN-Transformer) and used the same hyperparameters as in previous studies. By comparing GNNLink with these baseline methods, we aim to demonstrate the effectiveness and advantages of our approach in various settings.

1.2 Experimental setting

For the training of the GNNLink model, the training period is set to 200 and the learning rate is set to 0.005. We set the number of layers of the GNN to 2. The sizes of the hidden layers are set to 256 and 128, respectively. Although the above parameters are set empirically. However, the parameters of number of layers L and weight factor K in the GCN-based graphical encoder need to be parametrically analyzed in the following sections. The results provided in this subsection are based entirely on the predictions of the test set. All models in this paper are run on a computer configured with AMD Ryzen 7 4800U with Radeon Graphics 1.80 GHz, 8 cores and 16 GB RAM.

2 Supplementary Tables

Table. S1. Effect of noise level on network accuracy on four datasets with cell-type-specific networks.

Dataset	Noise	Metric	Method						
			GNNLink	GENELink[1]	GNE[2]	CNNC[3]	DeepDRIM[4]	GRN-Transformer[5]	GENIE3[6]
hESC	0	AUROC	0.83	0.77	0.64	0.62	0.42	0.47	0.46
		AUPR	0.49	0.41	0.3	0.19	0.13	0.11	0.14
	0.01	AUROC	0.81	0.75	0.6	0.61	0.42	0.47	0.45
		AUPR	0.48	0.38	0.29	0.18	0.13	0.1	0.1
	0.02	AUROC	0.8	0.74	0.6	0.61	0.4	0.46	0.43
		AUPR	0.48	0.38	0.27	0.17	0.12	0.09	0.1
	0.05	AUROC	0.78	0.7	0.58	0.57	0.34	0.41	0.38
		AUPR	0.43	0.31	0.23	0.16	0.1	0.09	0.07
hHEP	0	AUROC	0.78	0.79	0.76	0.6	0.46	0.5	0.43
		AUPR	0.7	0.64	0.63	0.42	0.34	0.31	0.29
	0.01	AUROC	0.78	0.78	0.76	0.6	0.43	0.49	0.41
		AUPR	0.67	0.64	0.62	0.42	0.31	0.3	0.29
	0.02	AUROC	0.76	0.74	0.72	0.58	0.4	0.47	0.4
		AUPR	0.65	0.6	0.6	0.41	0.27	0.29	0.28
	0.05	AUROC	0.73	0.7	0.68	0.56	0.38	0.42	0.37
		AUPR	0.59	0.58	0.57	0.38	0.25	0.26	0.22
mDC	0	AUROC	0.68	0.63	0.5	0.51	0.46	0.48	0.45
		AUPR	0.21	0.09	0.04	0.04	0.03	0.03	0.02
	0.01	AUROC	0.68	0.62	0.49	0.5	0.46	0.48	0.44
		AUPR	0.2	0.09	0.04	0.04	0.03	0.03	0.02
	0.02	AUROC	0.65	0.6	0.47	0.48	0.42	0.46	0.43
		AUPR	0.18	0.07	0.03	0.03	0.03	0.02	0.01
	0.05	AUROC	0.62	0.58	0.42	0.43	0.4	0.4	0.4
		AUPR	0.14	0.06	0.02	0.03	0.02	0.02	0.01
mESC	0	AUROC	0.8	0.76	0.76	0.69	0.5	0.5	0.46
		AUPR	0.74	0.74	0.61	0.44	0.42	0.46	0.29
	0.01	AUROC	0.79	0.76	0.74	0.69	0.5	0.48	0.46
		AUPR	0.7	0.71	0.6	0.43	0.42	0.45	0.29
	0.02	AUROC	0.76	0.73	0.72	0.68	0.48	0.48	0.42
		AUPR	0.68	0.67	0.58	0.4	0.38	0.43	0.26
	0.05	AUROC	0.73	0.7	0.69	0.62	0.42	0.43	0.4
		AUPR	0.67	0.65	0.55	0.35	0.32	0.4	0.21

Table. S2. Comparison of various positive instance ratios in training, validation, and test datasets for the hHEP dataset with the Cell-Type-Specific ChIP-seq network. To mitigate the bias introduced by random partitioning, each experiment was conducted 10 times, and the average AUROC and AUPR values were used as the final metrics.

Pos. Instances Ratio (Train/Val/Test)	Method	AUROC	AUPR
3:1:1	GNNLink	0.82	0.75
	GENELink[1]	0.85	0.68
	GNE [2]	0.8	0.67
	CNNC [3]	0.67	0.5
	DeepDRIM [4]	0.62	0.48
	GRN-Transformer [5]	0.58	0.5
	GENIE3 [6]	0.54	0.39
8:1:1	GNNLink	0.81	0.75
	GENELink [1]	0.85	0.68
	GNE [2]	0.79	0.67
	CNNC [3]	0.68	0.5
	DeepDRIM [4]	0.62	0.49
	GRN-Transformer [5]	0.57	0.51
	GENIE3 [6]	0.54	0.38
4:3:3	GNNLink	0.76	0.64
	GENELink [1]	0.71	0.62
	GNE [2]	0.72	0.6
	CNNC [3]	0.63	0.4
	DeepDRIM [4]	0.52	0.36
	GRN-Transformer [5]	0.49	0.4
	GENIE3 [6]	0.5	0.31

3 Supplementary Figures

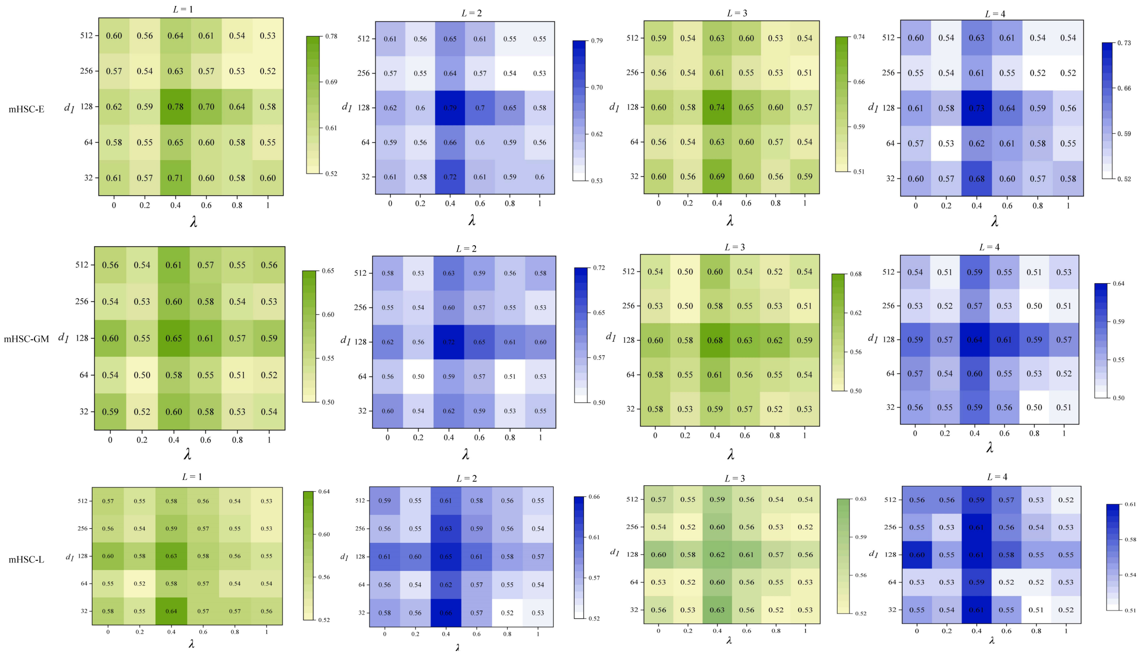

Fig. S1. Examining the impact of parameter combinations on predictive model performance and sensitivity analysis of the GNNLink model in the mHSC-L/GM/E Dataset with Cell-Type-Specific Networks. The GNNLink models were constructed using various parameter combinations for the mHSC-L/GM/E dataset with cell-type-specific networks, and the impact of these combinations on model performance was evaluated. To test the sensitivity of the GNNLink model to these parameters, we obtained the AUC values by varying these parameters. The color shading indicates the size of the value, each row corresponds to a dataset and each column represents the number of encoder layers (L). Abbreviations: AUC,Area Under the Curve.

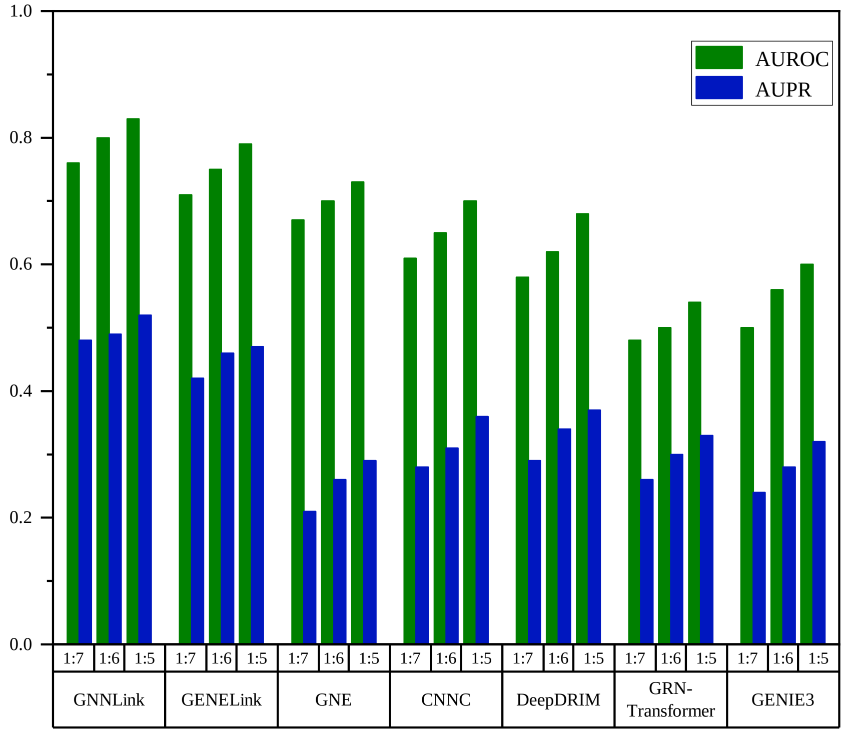

Fig. S2. Evaluating the impact of varying positive-to-negative sample ratios on model performance in mESC dataset with the LOF/GOF network. To investigate the effect of different positive-to-negative sample ratios on model performance, we sampled positive and negative links between gene pairs in the training and validation sets. The actual positive-to-negative ratio in the mESC dataset LOF/GOF network is approximately 0.187. We selected ratios of 1:5, 1:6, and 1:7 for positive-to-negative links. To mitigate the bias introduced by random sampling and partitioning, each experiment was conducted 10 times, with the average AUROC and AUPR values serving as the final metrics. Abbreviations:LOF/GOF, loss-of-function/gain-of-function.

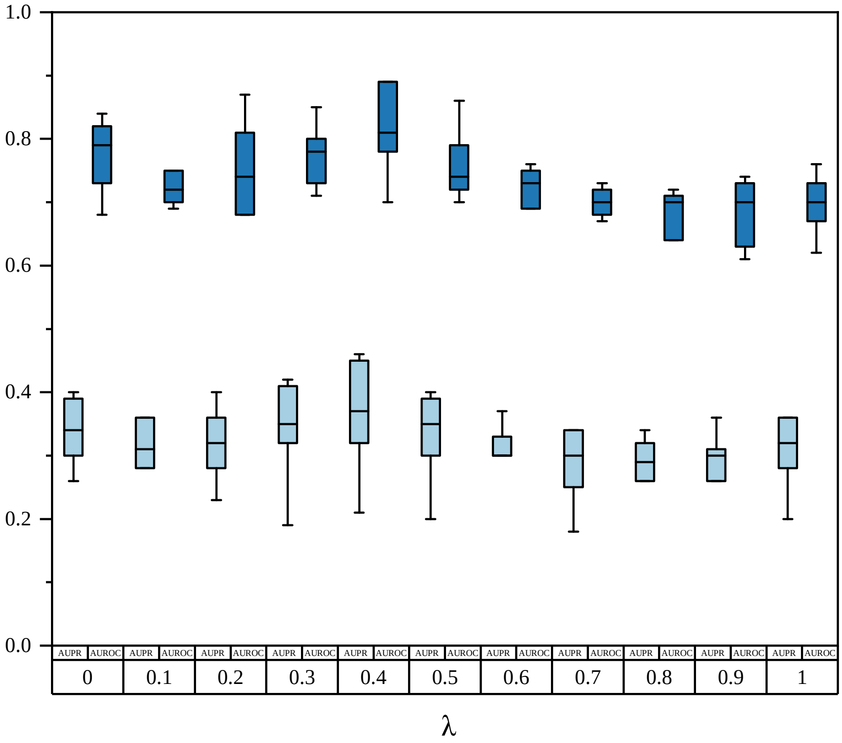

Fig. S3. Analyzing the impact of λ variations on GNNLink models across seven benchmark datasets with cell type-specific networks.

References

[1] Guangyi Chen and Zhi-Ping Liu. Graph attention network for link prediction of gene regulations from single cell rna-sequencing data. *Bioinformatics*, 38, 08 2022.

[2] Kishan Kc, Rui Li, Feng Cui, Qi Yu, and Anne R Haake. Gne: a deep learning framework for gene network inference by aggregating biological information. *BMC systems biology*, 13(Suppl 2):38, April 2019.

[3] Ye Yuan and Ziv Bar-Joseph. Deep learning for inferring gene relationships from single-cell expression data. *Proceedings of the National Academy of Sciences of the United States of America*, 116(52):27151—27158, December 2019.

[4] Jiaxing Chen, ChinWang Cheong, Liang Lan, Xin Zhou, Jiming Liu, Aiping Lu, William Cheung, and Lu Zhang. Deepdrim: a deep neural network to reconstruct cell-type-specific gene regulatory network using single-cell rna-seq data. 02 2021.

[5] Hantao Shu, Fan Ding, Jingtian Zhou, Yexiang Xue, Dan Zhao, Jianyang Zeng, and Jianzhu Ma. Boosting single-cell gene regulatory network reconstruction via bulk-cell transcriptomic data. *Briefings in bioinformatics*, 23(5):bbac389, September 2022.

[6] Vân Anh Huynh-Thu, Alexandre Irrthum, Louis Wehenkel, and Pierre Geurts. Inferring regulatory networks from expression data using tree-based methods. *PloS one*, 5(9):e12776, September 2010.

[7] Aditya Pratapa, Amogh Jalihal, Jeffrey Law, Aditya Bharadwaj, and T.M. Murali. Benchmarking algorithms for gene regulatory network inference from single-cell transcriptomic data. 05 2019.
